# Supplementary material for: Detection of Velogenic Avian Paramyxoviruses in Rock Doves in New York City, New York
Source: Microbiol Spectr. 2022 Mar 31;10(2):e02061-21. doi: 10.1128/spectrum.02061-21 (PMC9045282; doi:10.1128/spectrum.02061-21)
Supplement: SUPPLEMENTAL FILE 2 — Supplemental material. Download SPECTRUM02061-21_Supp_1_seq17.pdf, PDF file, 0.03 MB [file spectrum02061-21_supp_1_seq17.pdf]

Supplemental Table S1. Estimates of evolutionary divergence between isolates and closely related APMV-1 F protein sequences. The nucleotide distances between sequences are shown. Analyses were conducted using the Maximum Composite Likelihood model. This analysis involved 76 nucleotide sequences. Codon positions included were 1st+2nd+3rd+Noncoding. All ambiguous positions were removed for each sequence pair (pairwise deletion option). There were a total of 1656 positions.
